# Metabolic syndrome among marginalised school-going adolescents: a call for clarity

**DOI:** 10.1016/j.lansea.2024.100458

**Published:** 2024-08-03

**Authors:** Ajeet Singh Bhadoria, Archisman Mohapatra, Vineet Kumar Pathak, Mohan Kumar

**Affiliations:** a All India Institute of Medical Sciences – Rishikesh, Rishikesh, Uttarakhand, India; b The Generating Research Insights for Development (GRID) Council, Uttar Pradesh, India; c Amrita School of Medicine, Faridabad, Haryana, India; d KMCH Institute of Health Sciences and Research, Coimbatore, Tamil Nadu, India

With great interest, we read the article by Sharif and colleagues^1^ It is not easy to do a biomarker-based study on urban slum population in resource-constrained settings and we congratulate the team for the same. However, the study has certain serious concerns. As international readers, we could not get a context of the study—profile of the slum population, how this population differs from the urban non-slum population, school enrolment rate, why only two schools were selected and how do these compare with other slum populations, do these schools provide mid-day meals? The sample size of 689 participants was much inadequate given that the prevalence was much lower than the expected value of 50% used for the sample size calculation. We are also concerned about the sampling methods. There is no mention of refusal rates or even a comment on potential participation bias even if the number of children aged 11 and 12 years cumulatively accounted for just 1.8% of the total sample. Furthermore, the regression analysis looks inappropriate since the variables used to define metabolic syndrome were fitted into the model and declared as predictors.^2^, ^3^, ^4^, ^5^ The use of variables that define metabolic syndrome as predictors within the same model can lead to inappropriate conclusions, as these predictors should not simultaneously serve as components of the outcome variable. The use of vocabulary is also confusing. For example, the authors mention the ‘sensitivity’ of different criteria for ‘diagnosing’ metabolic syndrome (paragraph 2 of the results section).^1^ Several of the inferences discussed have been compared with studies that were not done in relatable contexts. We expect Sharif and colleagues to review these issues within their research article and suggest how readers should interpret and use the evidence it suggests.

## Contributors

Conceptualisation: ASB, AM, VKP, MK.

Data curation: VKP, MK.

Supervision: ASB, AM.

Writing—original draft: ASB, AM, VKP, MK.

Writing—review & editing: ASB, AM.

## Declaration of interests

None to declare.
